# Lysyl oxidase-like 2 processing by factor Xa modulates its activity and substrate preference

**DOI:** 10.1038/s42003-023-04748-8

**Published:** 2023-04-07

**Authors:** Huilei Wang, Alan Poe, Marta Martinez Yus, Lydia Pak, Kavitha Nandakumar, Lakshmi Santhanam

**Affiliations:** 1 Department of Biomedical Engineering, Johns Hopkins University School of Medicine, 733 N Broadway, Baltimore, MD 21205 USA; 2 Department of Chemical and Biomolecular Engineering, Johns Hopkins University Whiting School of Engineering, 3400 N Charles St, Baltimore, MD 21218 USA; 3 Department of Anesthesiology and CCM, Johns Hopkins University School of Medicine, 733 N Broadway, Baltimore MD, 21205 Baltimore, MD USA

**Keywords:** Cell biology, Cardiology

## Abstract

Lysyl oxidase-like 2 (LOXL2) has been identified as an essential mediator of extracellular matrix (ECM) remodeling in several disease processes including cardiovascular disease. Thus, there is growing interest in understanding the mechanisms by which LOXL2 is regulated in cells and tissue. While LOXL2 occurs both in full length and processed forms in cells and tissue, the precise identity of the proteases that process LOXL2 and the consequences of processing on LOXL2’s function remain incompletely understood. Here we show that Factor Xa (FXa) is a protease that processes LOXL2 at Arg-338. Processing by FXa does not affect the enzymatic activity of soluble LOXL2. However, in situ in vascular smooth muscle cells, LOXL2 processing by FXa results in decreased cross-linking activity in the ECM and shifts substrate preference of LOXL2 from type IV collagen to type I collagen. Additionally, processing by FXa increases the interactions between LOXL2 and prototypical LOX, suggesting a potential compensatory mechanism to preserve total LOXs activity in the vascular ECM. FXa expression is prevalent in various organ systems and shares similar roles in fibrotic disease progression as LOXL2. Thus, LOXL2 processing by FXa could have significant implications in pathologies where LOXL2 is involved.

## Abstract (web summary)

A role for Factor Xa in the proteolytic processing of LOXL2 is identified and the resulting processed LOXL2 changes affinity from collagen IV to collagen I and increasingly binds to LOX, suggesting that LOX could be recruited to the regions where cleaved LOXL2 is deposited.

## Introduction

Lysyl oxidase-like 2 (LOXL2) is a member of the lysyl oxidase (LOX) family of amine oxidases that catalyze the oxidative deamination of lysine resides on extracellular matrix (ECM) proteins, thus facilitating the formation of a stable and highly organized ECM^1^. A critical role for LOXL2 in cardiopulmonary diseases is emerging. LOXL2 is clearly important in vascular development as germline deletion of LOXL2 results in high perinatal lethality ascribed primarily to defects in the cardiovascular system^2^. In the cardiopulmonary system, LOXL2 mediates vascular stiffening in aging, cardiac interstitial fibrosis, pulmonary hypertension, and pulmonary fibrosis^3–6^. In addition, LOXL2 participates in the initiation and progression of a diverse set of diseases including fibrosis and cancer metastasis, which is consistent with the critical roles of LOXL2 in tubulogenesis, matrix assembly, and angiogenesis^3,5–10^. Thus, in conjunction with the prototypical LOX, LOXL2 has emerged as a key factor in the maintenance and pathologic remodeling of organ systems^11–13^.

Given LOXL2’s central role in driving ECM remodeling there is significant interest in developing an understanding of the mechanisms by which LOXL2 activity is regulated in cells and tissue. In this regard, LOXL2 processing in the extracellular space is of high interest. LOXL2 is secreted via the Golgi-pathway. While LOXL2 transcript predicts a protein of 87 kDa, Western blotting analysis has shown different forms of LOXL2: a full-length form of 95 kDa–105 kDa which is likely glycosylated, and proteolytically processed forms with the larger fragment noted at 50-63 kDa and the smaller fragment at ~35 kDa^14–17^. The two processed fragments have been identified as an N-terminal fragment of 317 amino acids comprised of SRCR domains 1–2 (smaller fragment) and a C-terminus fragment comprised of SRCR domains 3-4 and the LOX domain (larger fragment)^16,17^. This has resulted in the comparison of LOXL2 to the prototypical LOX as well as LOXL1, which are secreted as inactive pro-enzymes, and under specific cellular stress, processed by the pro-collagen C-proteinase bone morphogenic protein 1 (BMP-1) to release the active forms^18,19^. However, LOXL2 regulation by processing is distinct from LOX/LOXL1. First, LOXL2 is likely processed by serine proteases^16,17^ and not by BMP-1^20^. The specific identities of the proteases responsible for LOXL2 processing remains incompletely understood. Second, unlike LOX/LOXL1, LOXL2 processing is not necessary to unleash its catalytic function and full length LOXL2 exhibits catalytic function. The effect of processing on LOXL2’s catalytic activity remains unclear: while one study proposed that LOXL2 proteolytic processing is required for collagen IV (COLIV) crosslinking in the ECM^17^, another study found that processing did not significantly impact COLIV crosslinking activity^16^. Moreover, prior studies have not investigated if LOXL2 processing results in catalytic activity towards other collagens, particularly collagen I (COLI), or if processing alters LOXL2 substrate preference or affinity. Thus, the functional effect of LOXL2 processing is enigmatic, and its pathophysiological importance remains unclear. Therefore, in this study, we focused on identifying the protease that processes LOXL2, and determined the consequences of this processing on its activity and substrate preference in vascular smooth muscle cells.

## Results

### Identification of Factor Xa as a protease that processes LOXL2

**Fig. 1:**
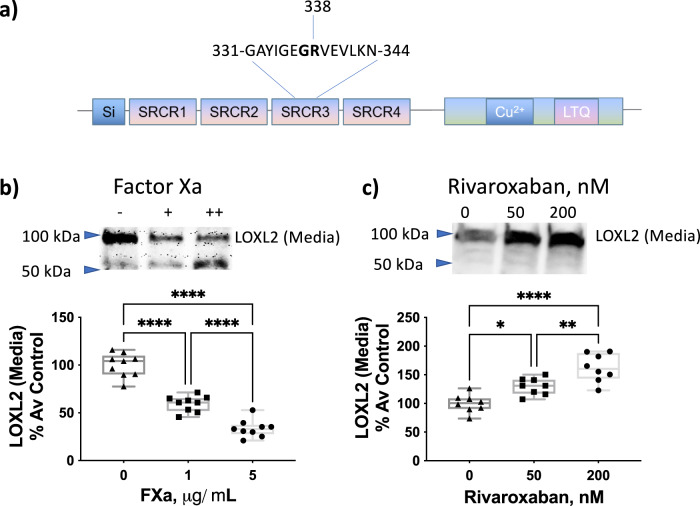
FXa processes soluble LOXL2 in cell-culture media. **a** Predicted site for FXa processing of LOXL2. **b** Representative western blot and densitometry analysis in the conditioned media of A7r5 cells showing increased processing of LOXL2 when cells are treated with increasing levels of exogenous FXa (0, 1, or 5 μg/ml) for 24 h (*n* = 9; ****P < 0.0001 by one-way ANOVA) and **c** Representative western blot and densitometry analysis in the conditioned media of A7r5 cells with inhibition of endogenous FXa using the FXa-specific inhibitor, rivaroxaban (0, 50, 200 nM) for 24 h shows the accumulation of the full-length LOXL2. (*n* = 8; mean ± SEM; *P < 0.05, **P < 0.01, ****P < 0.0001 by one-way ANOVA).

The prototypic LOX and LOXL1 are known to be secreted in a pro-form and require processing by BMP-1 to generate active LOX/LOXL1^18,19^. While processing of LOXL2 has been observed in western blotting in many previous studies^14,21–23^, the identity of the enzymes processing LOXL2 remains to be fully elucidated and was the focus of this study. We first experimentally verified that BMP-1 is not the protease responsible for LOXL2 processing, as has been previously reported^20^. Indeed, neither the addition of BMP-1 nor the use of BMP inhibitor UK 383367 altered LOXL2 processing. Next, we performed an in silico analysis of LOXL2 using ExPASy PeptideCutter with all listed available proteases and chemicals. FXa was identified as the only protease with less than 5 cleavage sites that cuts between SRCR 2 and 3. Importantly, a specific site at Arg-338 was predicted (Fig. 1a), matching the molecular weights of the two LOXL2 fragments observed in Western blots. Therefore, we hypothesized that FXa is a protease that cleaves LOXL2.

We next examined whether FXa can process LOXL2 using A7r5 cells that endogenously express LOXL2. The addition of exogenous, active Factor Xa to cell-culture media resulted in a marked decrease in full-length LOXL2 (~100 kDa) and an increase in the processed fragment (~65 kDa; Fig. 1b), detected using a monoclonal antibody targeting the C-terminus of LOXL2. Conversely, rivaroxaban (Rxb), a specific FXa inhibitor, attenuated processing of endogenous LOXL2 by endogenous FXa in a dose-dependent manner (Fig. 1c). Rivaroxaban preserved full-length LOXL2 and the processed smaller fragment was no longer detected. This confirms the findings of other studies that suggest LOXL2 is cleaved by serine proteases^16,17^, and identifies FXa as a specific candidate that can process LOXL2.

**Fig. 2:**
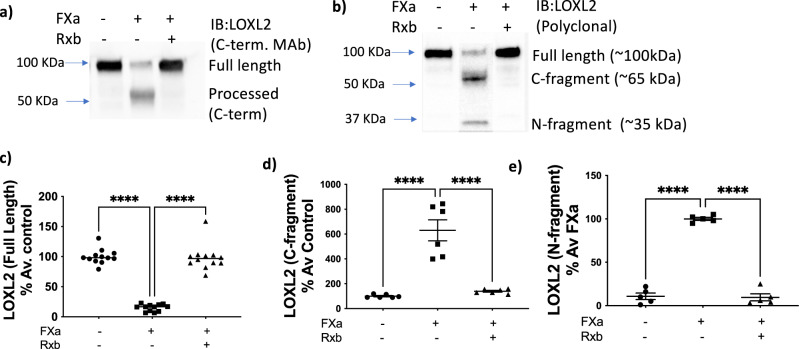
Cleavage of full-length LOXL2 (~100 kDa) by FXa produces a ~65 kDa and a ~35 kDa fragment. Representative western blots and densitometry analysis of in vitro processing of overexpressed LOXL2 in the conditioned media of A7r5 cells by FXa (1 μg/ml) in the presence and absence of rivaroxaban (Rxb, 100 nM) for 24 h. **a** A rabbit monoclonal antibody (Rab mAb) with epitope in the C-terminus of LOXL2 detects the full-length LOXL2 and a 65 kDa band. (*n* = 6–12, mean ± SEM; ****P < 0.0001 by one-way ANOVA). **b** A polyclonal antibody targeting LOXL2 detects the full-length LOXL2 and two processed bands (65 kDa and 35 kDa). Densitometry analysis of **c** full-length LOXL2 (~100 kDa), **d** C-terminus fragment (~65 kDa), and **e** N-terminus fragment (~35 kDa). (*n* = 5–6, mean ± SEM; ****P < 0.0001 by one-way ANOVA).

To further confirm processing of LOXL2 by FXa, we used an in vitro approach. A7r5 cells were transduced with adenovirus to overexpress C-terminus His-tagged full-length human LOXL2 (Supplementary Fig. 1). Endogenous FXa was detected in cell-culture media, and FXa abundance was not affected by AdLOXL2 treatment (Supplementary Fig. 1). Conditioned media was collected and treated with FXa (1 μg/ml) alone or FXa with rivaroxaban (100 nM). Western blotting using the C-terminus monoclonal antibody targeting LOXL2 revealed that incubation with FXa increased LOXL2 processing, illustrated in the loss of the full-length band and increase in the processed fragment, and Rivaroxaban completely blocked the processing of LOXL2 by FXa, and preserved full-length LOXL2 (Fig. 2a). Using a polyclonal anti-LOXL2 antibody, we identified 2 fragments (~65 kDa and ~35 kDa) upon FXa treatment (Fig. 2b). Again, full-length LOXL2 was preserved when FXa was inhibited with rivaroxaban (Fig. 2c–e). This confirms that FXa can process LOXL2 and produce processed forms of molecular weight similar to those seen in the literature and our previous Western blot analysis.

**Fig. 3:**
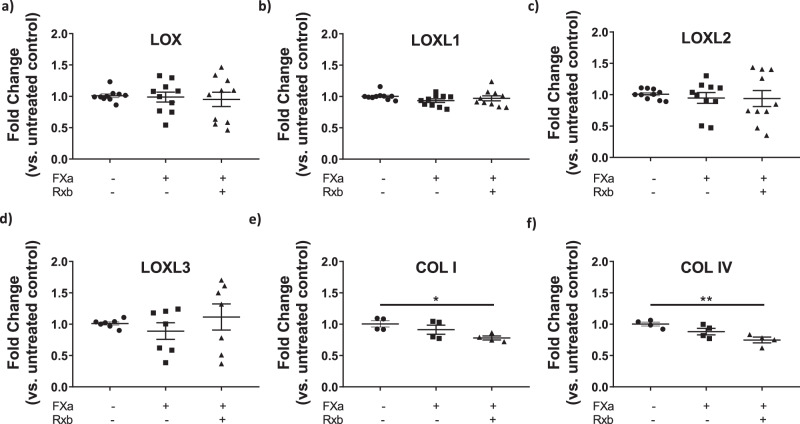
FXa does not alter gene expression of the LOX family. Gene transcription analysis by qPCR of **a** *LOX*, **b** *LOXL1*, **c** *LOXL2*, **d** *LOXL3*, **e** *COLI*, and **f** *COLIV*. Data are shown as fold-change in gene expression in A7r5 cells with or without 24 h of FXa (1 μg/ml) and rivaroxaban (Rxb, 100 nM) incubation, vs. average gene expression of untreated control A7r5s (*n* = 10 for *LOX, LOXL1,* and *LOXL2*, *n* = 7 for *LOXL3*, *n* = 4 for *COLI and COLIV*; *P < 0.05, **P < 0.01, by one-way ANOVA).

Next, we examined whether FXa regulates gene expression of the *LOX* proteins. No significant changes in the LOX family genes (*LOX, LOXL1, LOXL2*, and *LOXL3*) was noted with FXa in the presence or absence of rivaroxaban (Fig. 3a–d). We also examined transcription of *COLI* and *COLIV*. FXa treatment did not result in a meaningful change in *COLI* or *COLIV* transcripts. However, *COLI* and *COLIV* mRNA levels decreased in the presence of FXa + Rxb (Fig. 3e, f).

### Identifying LOXL2 cleavage site by FXa

**Fig. 4:**
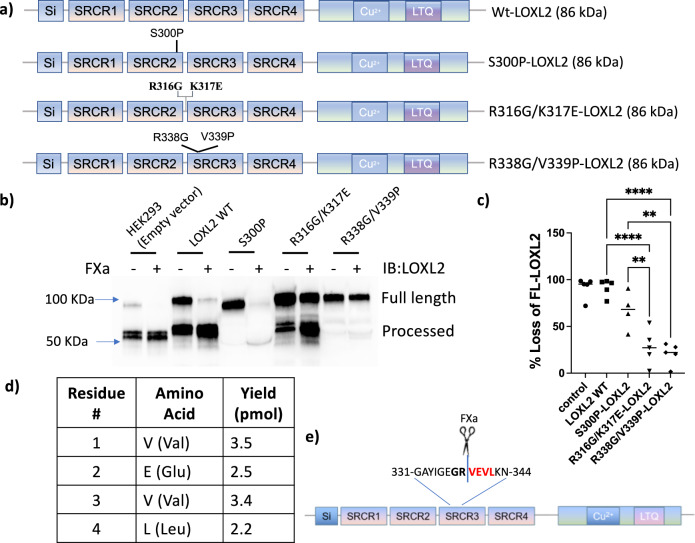
FXa processes LOXL2 at R338G. Conditioned media containing wild-type and mutant LOXL2 were incubated with FXa (1 μg/ml) for 2 h. **a** Schematic of LOXL2 mutants with predicted molecular weight in parenthesis. **b**, **c** Conditioned media containing similar levels of overexpressed LOXL2 mutants were incubated with and without FXa for 2 h. Proteins from conditioned media were then enriched with StrataClean Resin and LOXL2 processing was evaluated by western blotting. **b** Representative western blot and **c** densitometry analysis of FXa mediated processing of WT-LOXL2, S300P-LOXL2, R316G/K317E-LOXL2, and R338G/V339P-LOXL2. (*n* ***=*** 5; **P ***<*** 0.01, ****P ***<*** 0.0001 by one-way ANOVA). **d**, **e** C-terminal His-Tagged LOXL2 was incubated with FXa. The resulting C-terminus fragment was enriched using His-Talon beads and sequenced by Edman degradation. **d** Table showing the first 4 amino acids of the processed (~65 kDa) C-terminus fragment of LOXL2 and **e** schematic depicting the FXa processing site of LOXL2.

To identify the location of LOXL2 processing by FXa, we created three LOXL2 mutant constructs (Fig. 4a), (1) R338G/V339P-LOXL2, (2) R316G/K317E-LOXL2, and (3) S300P-LOXL2, and identified which of these were refractory to processing by FXa. The choice of mutated primary sequence was guided by prior findings showing that the substrate residue N-terminal to the cleavage site (P1) largely determines the specificity of serine proteases. In silico digestion of LOXL2 by FXa suggests a primary potential cut site at Arg-338, since FXa is highly specific for cleavage at Arg in position P1 and Gly in position P2^24–26^. To examine the putative cut site at Arg-338, P1 was mutated to disrupt the X-X-Gly-Arg recognition sequence of FXa, and P1’ was also mutated as prior studies show that FXa cleavage is prohibited with proline at P1’ position^27,28^. Thus, we generated a R338G/V339P double mutant to remove this putative processing site by FXa. Prior studies have reported Lys-317 as the cleavage site of LOXL2 processing^16,17^, and indicated Arg-X-Arg-Lys/Arg as the recognition sequence with a strong preference for Arg over Lys at P1. Because FXa is also known for its promiscuity in cryptic cleavage sites, and can cut at a seemingly arbitrary lysine or arginine^29,30^, we also included Lys-317 as a putative cut site in our study. To test the Lys-317 cleavage site, we generated a R316G/K317E-LOXL2 double mutant to disrupt both recognition sites at P1 and P2, as this was shown to be necessary in a prior study^16^. Finally, S300 was chosen as a serine site that is not predicted to be targeted by proteases, to serve as null control.

To identify the specific processing site targeted by FXa, HEK293 cells were transfected to transiently overexpress WT-LOXL2, S300P-LOXL2, R316G/K317E-LOXL2, and R338G/V339P-LOXL2 (Fig. 4a). All mutants were expressed and secreted normally. Cell-culture medium containing overexpressed WT or mutant LOXL2 protein was collected and incubated with or without FXa (1 μg/ml) for 2 h, and LOXL2 processing was evaluated by Western blotting (Fig. 4b). The loss of LOXL2 full length band with FXa treatment was quantified with respect to the paired FXa untreated control for each mutant/wild-type LOXL2 (Fig. 4c). FXa processed >80% of endogenous LOXL2 (HEK293; empty vector control) and overexpressed WT-LOXL2. Interestingly, the S300P-LOXL2 mutant was readily processed and degraded by FXa, as evidenced by the complete loss of both the full-length and the processed bands in the FXa-treated condition. While R316G/K317E-LOXL2 was partially protected from FXa processing, a significant level of processed band was observed. On the other hand, R338G/V339P-LOXL2 was completely refractory to processing by FXa; the modest decrease (~20%) in the full-length LOXL2 noted in the presence of FXa is likely the processing of endogenous LOXL2 expressed by HEK293 cells. These experiments confirm Lys-317 and Arg-338 as targets of FXa processing. To further determine the processing site, we purified the processed 65kDa C-terminal His-tagged fragment using affinity purification and determined the N-terminal sequence of this fragment by Edman Degradation analysis (Proteomics Core, Johns Hopkins University). This approach revealed the first four N-terminal amino acids of the 65 kDa processed fragment to be VEVL (Fig. 4d). Thus, FXa cleaves after the arginine residue R338 of LOXL2 (Fig. 4e).

### LOXL2 processing regulates ECM cross-linking activity and substrate preference

**Fig. 5:**
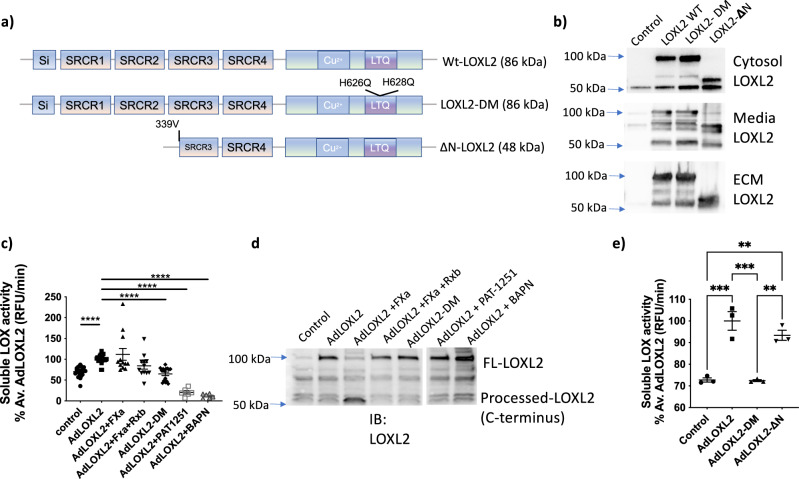
Processing by FXa does not influence the activity of soluble LOXL2. **a** Schematic of LOXL2 mutants used with predicted molecular weights in parenthesis and **b** representative Western blotting image of HEK293 cells overexpressing WT-LOXL2, LOXL2-DM, and ΔN-LOXL2 by transfection in the cytosol, conditioned media, and ECM. Equal amounts of protein were loaded for the cytosol, and a normalized volume of ECM and conditioned media proteins based on the cytosolic protein concentration were loaded on gels. **c** LOX activity in conditioned media measured by Amplite Fluorimetric LOX assay kit under indicated conditions. LOX activity was normalized to the average of AdLOXL2 condition. Data are shown as mean ± SEM (*n* ≥ 6, ****P < 0.0001 by one-way ANOVA). **d** Representative Western blot of LOXL2 expression in the cell-culture media used for the Amplex Red activity assay. **e** LOX activity in the cytosolic fraction measured by Amplite Fluorimetric LOX assay kit under indicated conditions. LOX activity was normalized to the average of AdLOXL2 condition. Data are shown as mean ± SEM (*n* = 3 **p < 0.01, ***p < 0.001 by one-way ANOVA).

Since the cleavage site at Lys-317 has been previously described, we next focused on studying the functional consequences of processing at the newly discovered cleavage site Arg-338 on LOXL2’s catalytic function. The primary question regarding LOXL2 processing is whether or not processing regulates LOXL2 catalytic function. To address this question, we created ΔN-LOXL2 resembling processed LOXL2 by deleting the first 338 amino acids on the N-terminus and enzymatically inactive LOXL2-DM (H626/628Q double mutant) as a negative control for activity assays (Fig. 5a). LOXL2-DM and ΔN-LOXL2 expressed well, and were secreted to the extracellular space. WT-LOXL2 and LOXL2-DM were identified in the cytosol, media, and ECM (Fig. 5b). Interestingly, ΔN-LOXL2 was more robustly identified in the cytosol and ECM, and did not accumulate in the conditioned media (Fig. 5b).

We first determined if FXa processing regulates catalytic activity of soluble LOXL2 recovered from the media and the cytosol. A7r5 cells were transduced with adenovirus to overexpress WT-LOXL2 (AdLOXL2). Cell-culture media was collected and incubated with or without FXa and rivaroxaban. Conditioned media from untransduced cells, AdLOXL2-DM, and AdLOXL2 media treated with LOXL2 inhibitor PAT-1251 or pan-LOX inhibitor BAPN were used as controls. ΔN-LOXL2 overexpression media was not investigated due to the low levels of overexpressed ΔN-LOXL2 in the media. Total LOX activity in the media was detected using the Amplite Fluorimetric LOX assay kit. As expected, WT-LOXL2 overexpression media displayed higher catalytic activity when compared with untransduced controls (Fig. 5c). Western blotting showed similar levels of wild-type, processed, and mutant LOXL2 in the media used for activity assays (Fig. 5d). While activity was attenuated by the specific LOXL2 inhibitor PAT-1251 or pan-LOX inhibitor BAPN, treatment with FXa did not lead to any change (increase or decrease) in total LOX activity. These findings are consistent with a recent study in which the structure of LOXL2 was solved at 2.4-Å resolution and showed that the SRCR domains point outwards from LOXL2^31^, suggesting that cleavage of the SRCR1-2 is unlikely to change LOXL2 catalytic function directly. Indeed, catalytic activity of processed LOXL2 towards COLIV is also reported to be similar to unprocessed (full length) LOXL2 in vitro^16^. We next evaluated activity of soluble LOXs obtained from the cytosol (Fig. 5e) to determine the activity of soluble ΔN-LOXL2. Untransduced cells (control), WT-LOXL2 and LOXL2-DM were used as controls. Catalytic activity of soluble ΔN-LOXL2 was similar to that of soluble full length LOXL2.

**Fig. 6:**
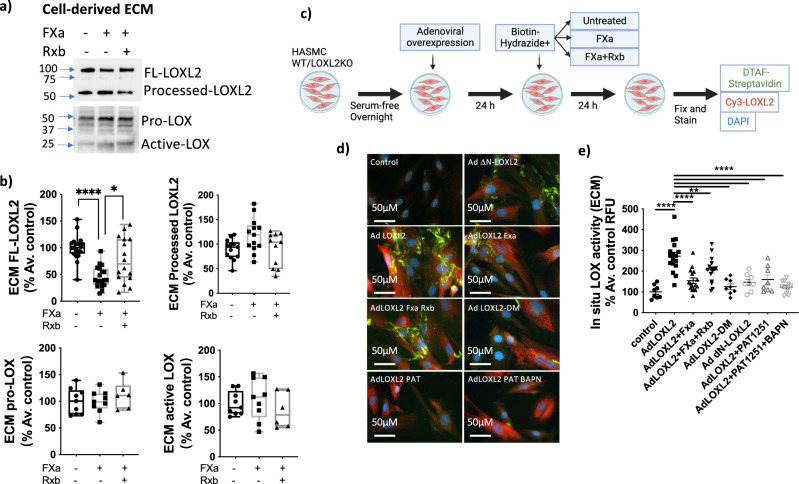
LOXL2 processing by FXa reduces total LOX activity in the cell-derived ECM. **a** Representative Western blot and **b** densitometry analysis showing full-length and processed LOXL2 and prototypical LOX in the cell-derived ECM with and without FXa treatment in the presence or absence of rivaroxaban (*n* ≥ 6; mean ± SEM; *P < 0.05, ****P < 0.0001 by one-way ANOVA). **c** Schematic depicting the in situ activity assay protocol (Created using Biorender). **d** Representative confocal microscopy images of in situ LOXs activity (green), LOXL2 (red), and nuclei (DAPI, blue) for the following conditions: (1) control A7r5s cells, (2) ΔN-LOXL2 overexpression, (3) wild-type LOXL2 overexpression (4) LOXL2 overexpression with FXa incubation (1 μg/ml), (5) LOXL2 overexpression with FXa (1 μg/ml) and rivaroxaban (50 nM) incubation, (6) catalytically inactive LOXL2-DM overexpression, (7) LOXL2 overexpression + LOXL2-specific inhibitor PAT-1251 (10 µM), (8) LOXL2 overexpression + inhibitors BAPN (10 µM) + PAT-1251 (10 µM). (Scale bar = 50 μm). **e** Activity signal in each IF image was converted to mean gray value shown in bar graph (*n* ≥ 8; mean ± SEM; **P < 0.01, ****P < 0.0001 by one-way ANOVA).

Interestingly however, prior studies have shown that LOXL2 processing augments COLIV deposition by cultured PFHR9 cells^17^. Taken together, these results suggest that while soluble LOXL2 activity is not modulated by processing, in the ECM, where catalytic activity of LOXL2 is most relevant, LOXL2 activity could be regulated by processing. To test this, we exposed A7r5 cells to FXa for 48 h with or without rivaroxaban. Western blotting showed loss of full length LOXL2 in the ECM (Fig. 6a, b). Prototypical LOX expression remained unchanged in the ECM in the presence of FXa and was detected only in the pro-form, suggesting little to no contribution from the prototypical LOX to overall LOXs activity. We next measured total LOX activity in the cell-derived ECM to better capture the enzymatic activity in situ to deposit cell-derived matrix^32^. Intermediates generated by LOXs catalytic activity on ECM proteins were biotinylated with biotin hydrazide (BHZ), labeled with fluorescein-conjugated streptavidin, and analyzed by confocal microscopy (Fig. 6c). LOXL2 overexpression (AdLOXL2) resulted in higher activity signal vs. untransduced condition (control); this was attenuated in the presence of FXa, and recovered by rivaroxaban (Fig. 6d, e). LOXL2 processing was verified by Western blotting of the conditioned media, where FXa treatment resulted in significant loss of the full length LOXL2 and rivaroxaban preserved full length LOXL2 (Supplementary Fig. 2). ∆N-LOXL2 overexpression showed similar levels of activity as AdLOXL2+FXa. Overexpression of the inactive LOXL2-DM as well as LOXL2 inhibition with PAT-1251 showed significantly lower activity signal when compared with that of full-length wild-type LOXL2.

We next sought to understand whether processing affects LOXL2 substrate preference and identify relevance to human arterial aging, where LOXL2 activation in the aortic smooth muscle cells plays a significant role^3^. To this end, we examined the consequences of FXa processing of endogenously expressed LOXL2 in human aortic smooth muscle cells (HASMC). We determined in situ interactions of processed and full-length LOXL2 with collagen I and IV, two of the most abundant ECM protein substrates of LOXs in the vasculature, by proximity ligation assay (PLA) and by co-immunofluorescent staining for LOXL2 and ECM substrates.

**Fig. 7:**
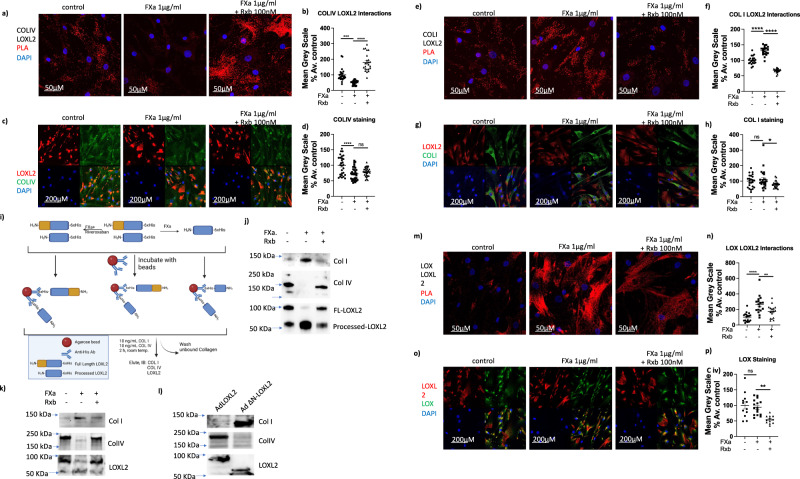
LOXL2 processing regulates its substrate preference and direct interactions with LOX in vascular smooth muscle cells. **a**–**d** Representative images and mean grayscale quantitation of LOXL2-COLIV interactions by **a**, **b** proximity ligation assay (PLA) (red = PLA signal of LOXL2-COLIV interaction; blue = nuclei) and **c**, **d** immunofluorescence (red = LOXL2, Green = COLIV, blue = nuclei). **e**–**h** LOXL2-COLI interactions by **e**, **f** PLA (red = LOXL2-COLI interaction; blue = nuclei) and **g**, **h** immunofluorescence (red = LOXL2, Green = COLI, blue = nuclei). **i**, **j** Interaction of processed and full-length LOXL2 with COLI and COLIV was investigated using purified proteins in vitro **i** schematic of the binding assay using immobilized full-length and processed forms of a C-terminus 6xHis-tagged LOXL2. (Created using BioRender). **j** Western blot showing COLI, COLIV, and LOXL2 eluted from the beads. Blot is representative of 4 independent experiments. **k**, **l** Representative Western blots showing COLI, COLIV, and LOXL2 in the cell-derived matrix prepared by de-cellularization of HASMCs cultured under the following conditions: **k** ECM from cells that were incubated with FXa with or without rivaroxaban for 24 h and **l** ECM from cells overexpressing full-length LOXL2 or ΔN-LOXL2 for 24 h. Blots are representative of 4 independent experiments. **m**–**p** Representative images and mean grayscale quantitation of LOXL2-LOX interactions by **m**, **n** PLA (red = PLA signal of LOXL2-LOX interaction; blue = nuclei) and **o**, **p** immunofluorescence (red = LOXL2, Green = LOX, blue = nuclei). Confocal images are representative of 3 separate experiments. All PLA signal and protein expression signals are quantified by mean grayscale and normalized to % average of controls (*n* = 24, mean ± SEM; *P < 0.05, **P < 0.01, ***P < 0.001, ****P < 0.0001 by one-way ANOVA).

Human aortic smooth muscle cells (HASMCs) were seeded on coverslips and incubated with FXa with and without rivaroxaban for 24 h and fixed. PLA showed that LOXL2 processing by FXa reduced COL IV-LOXL2 interactions by half when compared with controls. Accumulation of full-length LOXL2 by adding rivaroxaban increased COLIV-LOXL2 interactions by 80% (Fig. 7a, b), despite a decrease in COLIV mRNA expression (Fig. 3). Immunofluorescent staining of COLIV in the cell-derived ECM showed that deposition of COLIV reduced with FXa processing. Surprisingly, however, this was not sufficiently reversed when FXa was inhibited by rivaroxaban (Fig. 7c, d). Interestingly, COLI-LOXL2 interactions showed the opposite effect with LOXL2 processing by FXa. Both PLA and co-immunofluorescent staining revealed that LOXL2 interactions with COLI increased by 30% in the presence of FXa, and decreased by ~50% when FXa was inhibited by rivaroxaban (Fig. 7e–h). These differences in substrate binding are not due to degradation or processing of COLIV or COLI by FXa, as verified by incubation of purified COLI and COLIV with FXa (24 h at 37 °C; Supplementary Fig. 3a). LOXL2 processing by FXa was verified by Western blotting of the cell-culture supernatant **(**Supplementary Fig. 3b).

We next used an in vitro assay to evaluate the binding of full-length and processed LOXL2 with COLI and COLIV, wherein immobilized LOXL2 was incubated with a 1:1 (w/w) mixture of COLI and COLIV (Fig. 7i). COLI binding was elevated and COLIV binding was diminished with processed LOXL2 (FXa treated condition) when compared with untreated control and rivaroxaban treated samples, both of which had a higher level of full-length LOXL2 (Fig. 7j). We finally determined if LOXL2 processing leads to a shift in COLI vs. COLIV content in the cell-derived matrix using two approaches: 1) HASMCs were treated with FXa in the presence or absence of rivaroxaban; untreated cells served as controls and 2) Full-length LOXL2 or ΔN-LOXL2 was overexpressed in HASMCs. After 24 h, samples were decellularized and the ECM was recovered for Western blotting. FXa treatment led to higher COLI and lower COLIV in the cell-derived ECM when compared with untreated controls; this was reversed by rivaroxaban (Fig. 7k). Similarly, a higher amount of COLIV was noted in the ECM of cells overexpressing full-length LOXL2, and a higher level of COLI was observed in the ECM of cells overexpressing ΔN-LOXL2 (Fig. 7l).

### LOXL2 processing regulates LOX-LOXL2 interaction

In several disease processes, increase in LOX and LOXL2 activation are noticed to occur together. We therefore next evaluated if LOXL2 recruits LOX to the ECM. Western blotting showed that LOXL2 knockout in the HASMC results in significant loss of LOX in the cell-derived ECM (Supplementary Fig. 4). In the presence of FXa, LOX-LOXL2 interactions increased (Fig. 7m, n), despite similar levels of LOX expression (Fig. 7o, p). This suggests that processed LOXL2 recruits prototypical LOX to the ECM. Western blotting results indicate that LOX is in the pro-form and not the processed form in resting cells. This suggests that in the vasculature, LOX recruitment by LOXL2 might be able to compensate for overall decreased activity of processed LOXL2 under pro-fibrotic conditions where BMP-1 is present to process and activate LOX.

### FXa expression is prevalent in several organ systems

**Fig. 8:**
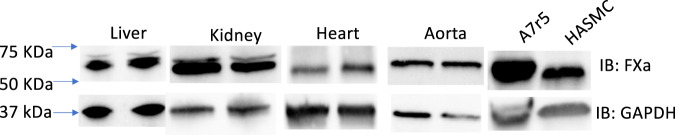
FXa is expressed in various organ systems. Representative western blots showing FXa expression in homogenates of organs and cells. GAPDH was used as the loading control. (*n* = 6 mice; 3 male and 3 female, ages 12–14 weeks old).

We finally determined if LOXL2 processing by FXa has in vivo pathophysiological significance. Though FXa was traditionally believed to be only synthesized in the liver^33,34^, ectopic expression of FXa has been found in various tissues, including heart, lung, brain, and cancerous tissues^35–38^. In this study, we also confirmed FXa expression in mouse heart and vascular smooth muscle cells, namely A7r5s and HASMCs as well as liver, kidney, heart, and aorta (Fig. 8). Thus, in the context of cardiovascular diseases, FXa expression in the heart and vasculature can represent a regulatory mechanism for LOXL2.

## Discussion

LOXL2 is an attractive target in cardiovascular aging and in a diverse set of disease processes owing to its fibrogenic role^3,4,7,11,14,39–41^. Understanding the mechanisms by which LOXL2 itself is regulated can yield insights towards the development of therapies. In this regard, proteolytic processing of LOXL2 is a mechanism of regulation that remains to be fully elucidated. Prior studies showed that serine proteases cleave LOXL2 at K317/A318, which remove the first two SRCR domains. Okada et al.^16^ identified PACE4 as a protease responsible for LOXL2 cleavage. In this study, we identified FXa as another major catalyst of LOXL2 processing, with an additional cleavage site at R338/V339. Previous studies showed that mutations at multiple Arg residues near Lys-317 were necessary to block LOXL2 processing, which was supported in our study as R316G/K317E-LOXL2 was protected from processing. On the other hand, S300P-LOXL2 was readily degraded in the presence of FXa, and the processed band was also eliminated, suggesting S300 is required LOXL2 protein stability. Post-translational modifications, such as *O*-glycosylation may be important, and the specific underlying mechanism(s) remain to be elucidated. Finally, R338G/V339P-LOXL2 was resistant to processing by FXa. This newly identified processing site sits in SRCR3, indicating that additional forms of processed LOXL2 exist, and may have functional differences due to the partial absence of SRCR3.

Similar to prior studies^16,17^, no significant differences were noted in the amine oxidase activity of soluble processed forms of LOXL2, as determined by the Amplite Fluorimetric Lysyl Oxidase Assay that measures H_2_O_2_ co-product released during the amine oxidase catalytic cycle. However, an in situ LOX activity assay that uses endogenous cell-derived ECM proteins as substrates showed that FXa processing reduced the overall LOXs activity in the ECM of vascular smooth muscle cells. This indicates that although processed LOXL2 has similar enzymatic activity as full-length LOXL2 with small, soluble substrates, its interactions with various ECM substrates might have changed. For example, the pro-domains of LOX and LOXL1 are known to play a significant role in substrate recognition and are required for the recruitment of both enzymes onto elastic fibers by mediating interactions with tropoelastin^42^. Similarly, the SRCR domains have been found to have a scaffolding role in recruiting and anchoring ECM substrates for catalysis^43^. Thus, the release of SRCR domains upon LOXL2 processing may disrupt or shift LOXL2’s substrate preference. Results from the PLA and in vitro binding assays support this by showing that LOXL2 interactions with COLI and COLIV are perturbed by LOXL2 processing (Fig. 7). In the HASMCs, LOXL2 processing shifted substrate preference away from COLIV and towards COLI. This result is in contrast to the previous studies in which PFHR9 cells were used to interrogate the role of LOXL2 processing: Okada et al.^16^ observed that processing only modestly increased LOXL2 cross-linking of COLIV, whereas Lopez-Jimenez et al.^17^ concluded that processing is necessary for COLIV crosslinking. Both studies used an in vitro assay to detect COLIV 7 S domains in ECM deposited from PFHR9 cells. These differences could be due to the disparities between cell types, as vascular smooth muscle cells (VSMC) in culture deposit a smaller amount of matrix than do fibroblasts and other epithelial or cancer cell lines, and the matrix deposited by each cell type is distinct in composition, mechanical properties, and structure. Taken together, these findings suggest that LOXL2 regulation by proteolytic processing is contextual, and dependent on the organ system. The production of tissue-specific types of collagen and the alteration of their colocalization with full-length versus processed LOXL2 would define the cell-type or organ-specific role for LOXL2 in homeostasis and disease. In this study, we investigated COLIV and COLI as examples of abundant ECM proteins in the vascular media. How LOXL2 processing shifts its binding of other substrates such as tropoelastin and COLIII that are also highly expressed in the vascular media remains to be investigated. This will have significant implications in vascular physiology and pathology, as VSMC phenotype and function are highly dependent on matrix composition, organization/architecture, and stiffness.

In this study, we also reported the colocalization and direct interaction of LOX and LOXL2. LOX and LOXL2 have often been observed to be upregulated together in a number of pathologies^4,44,45^, yet no study has clearly shown that the LOX family proteins interact with one another in any functional way. The PLA results showed that LOX and LOXL2 are highly colocalized, and their interactions increased significantly in the presence of FXa. A limitation of this study is that the specific domains of LOXL2 that bind LOX were not identified, and thus the mechanism by which LOXL2 recruits LOX to the ECM remains unknown. Nevertheless, the recruitment of pro-LOX to the ECM by LOXL2 processing sets the stage for elevated total LOX activity in the ECM in the presence of pro-fibrotic signals that activate BMP-1 and the subsequent processing and activation of LOX.

Besides the hemostatic role of FXa in the blood coagulation cascade, FXa is also known to induce complex signaling events and cellular responses that contribute to fibro-proliferative pathology, including fibrosis and tumor metastasis^46,47^. FXa promotes the proliferation and migration of SMCs and enhances the secretion of fibronectin, collagen, and TGF-β in fibroblasts^48–51^. FXa mediates intracellular signaling in many cell types, including macrophages, endothelial cells, fibroblasts, vascular smooth muscle cells, and cancer cells^50–56^. The effect of FXa signaling is highly specific according to the tissue and cell type. Inhibition of FXa in mouse models has shown protective effects in liver and renal fibrosis^57,58^. LOXL2 has also been implicated in the development of various fibrotic diseases and cancer due to its ability in intracellular signaling of epithelial-mesenchymal transition as well as ECM remodeling. As FXa and LOXL2 share similar roles in fibrotic disease progression and are promising therapeutic targets, it is of high interest to better understand whether and how they cooperate in cell signaling and ECM remodeling. In conclusion, our studies identify FXa as a protease responsible for LOXL2 processing in vascular smooth muscle cells. While it has been difficult to target LOXL2 specifically, the availability of safe and specific FXa inhibitors and the expression of both LOXL2 and FXa in several organs indicate that targeting FXa in vivo could serve as a surrogate route to reduce LOXL2-mediated collagen I deposition in the vasculature and potentially, to target fibrosis in other organs.

## Methods

### Reagents

Activated Factor X (FXa) was purchased from New England Biolabs. Rivaroxaban was purchased from Santa Cruz. For Western blotting and immunofluorescence staining, the following antibodies were used: LOX rabbit polyclonal (ThermoFisher PA1-46020), LOXL2 rabbit monoclonal C-terminal (Abcam ab179810), LOXL2 polyclonal (Abcam ab197779), COLI monoclonal antibody (Invitrogen MA1-26771), COLIV rabbit polyclonal (Assay Biotech C0157), Factor X/Xa polyclonal antibody (Invitrogen PA5-102412), GAPDH mouse monoclonal (Novus Bio NB300221), goat anti-mouse IgG (H + L)-HRP conjugate (Biorad 1706516), AffiniPure goat anti-rabbit IgG (H + L)-HRP conjugate (Jackson ImmunoResearch 111035144), Cy5 AffiniPure Goat Anti-Rabbit IgG (H + L) (Jackson ImmunoResearch, 111175144), Alexa Fluor 568 Goat anti-Rabbit IgG (H + L) Secondary Antibody (Invitrogen, A-11011), Alexa Fluor 488 Donkey anti-Mouse IgG (H + L) Secondary Antibody (Invitrogen, A-21202) and fluorescein (DTAF) streptavidin (Jackson ImmunoResearch, 016010084). An adenovirus to overexpress C-terminal His/mCherry-tagged LOXL2 was purchased from Vector Biolabs. BAPN (β-aminopropionitrile) was purchased from Sigma-Aldrich and dissolved in PBS. PAT-1251 (IUPAC name: 3-((4-(aminomethyl)−6-chloropyridin-2-yl)oxy)phenyl)((3 *R*,4 *R*)−3-fluoro-4-hydroxypyrrolidin-1-yl)methanone, CAS# 2098884-53-6) was obtained from Sundia Meditech Co.Ltd, and prepared in 0.5% methyl cellulose in DI water^59^. COLI was purchased from Sigma-Aldrich. COLIV solution was purchased from Santa Cruz. All other reagents were of the highest purity commercially available.

### In silico analysis of LOXL2 protein

The primary amino-acid sequence of human LOXL2 was analyzed using ExPASy Protein Cutter to identify putative protease digestion sites.

### Cell culture

A7r5 rat thoracic artery smooth muscle cells (ATCC) and HEK293 cells (ATCC) were cultured in Dulbecco’s modified Eagle’s medium (DMEM) supplemented with 10% fetal bovine serum (FBS, Thermo Fisher) and 1% antibiotic-antimycotic (Thermo Fisher). Human aortic smooth muscle cells (HASMCs, ThermoFisher) were cultured in smooth muscle cell media (ScienCell) containing 2% fetal bovine serum, SMC-growth supplement, and antibiotic-antimycotic. LOXL2-depleted HASMC cells were generated by targeting the *LOXL2* gene by CRISPR-Cas9 gene editing as previously described^32^. All cells were cultured in humidified incubators with 5% CO_2_ at 37 °C.

### LOXL2 cloning, mutagenesis, and adenoviral vectors

Human LOXL2 cDNA was obtained from the Arizona State University plasmid repository in pDONR221 plasmid (HsCD00041668) as a template. 6xHis-tagged LOXL2 was constructed by PCR using untagged human LOXL2 as template. LOXL2 626/628Q double mutant (LOXL2-DM), which is enzymatically inactive due to mutations of two histidines H626/628 critical for catalytic activity^31^, was generated using the QuikChange site-directed mutagenesis kit (Thermo Fisher). ΔN-LOXL2, equivalent to the C-terminus fragment of LOXL2 processed by FXa, was created by deleting the first 338 amino acids. Adenoviruses encoding C-terminus 6xHis-tagged full-length LOXL2 and LOXL2-DM and C-terminus V5-tagged ΔN-LOXL2 were constructed by LR recombination with destination vector pAd/DEST. The pAd/DEST vectors were digested with PacI, ethanol precipitated, and transfected into HEK293 cells. After cytopathic effect, adenoviruses were collected and purified via repeated freeze-thaw cycles and a Millipore adenovirus purification Kit.

To verify the site at which FXa cleaves LOXL2, three mutant LOXL2 constructs in pEZY plasmids were created with single or double mutations at putative sites: amino acid 300, 316–317, and 338–339, using QuikChange site-directed mutagenesis kit. S300P-LOXL2,R316G/K317E-LOXL2, R338G/V339P-LOXL2 were generated. Primers used for cloning and mutagenesis are shown in Table 1. LOXL2 mutations were verified by Sanger sequencing.

**Table 1:** PCR primers used for cloning and site-directed mutagenesis.

LOXL2	Fw	TTG TAT TTC CAG GGC GAG AGG CCT CTG TGC
	Rv	CAA GCT TCG TCA TCA CAA CTG CGG GGA CAG
LOXL2-DM	Fw	ATC TGG CAC GAC TGT CAA AGG CAA TAC CAC AGC ATG
	Rv	CAT GCT GTG GTA TTG CCT TTG ACA GTC GTG CCA GAT
ΔN-LOXL2	Fw	GGGG ACA AGT TTG TAC AAA AAA GCA GGC TTC ACC ATG GTG GAG GTG CTC AAA AATG
	Rv	GGGG AC CAC TTT GTA CAA GAA AGC TGG GTT CTG CGG GGA CAG CTG GTT GTT
S300P-LOXL2	Fw	CTA CCG GCC GTG GTG CCT TGT GTG CCT GGG CAG
	Rv	CTG CCC AGG CAC ACA AGG CAC CAC GGC CGG TAG
R316G/K317E-LOXL2	Fw	CCC TCG AGA TTC GGG GAA GCG TAC AAG CCA GAG
	Rv	CTC TGG CTT GTA CGC TTC CCC GAA TCT CGA GGG
R338G/V339P-LOXL2	Fw	TAC ATC GGG GAG GGC GGC CCG GAG GTG CTC AAA AAT
	Rv	ATT TTT GAG CAC CTC CGG GCC GCC CTC CCC GAT GTA

### LOXL2 protein processing

A7r5 cells were seeded at 80% confluence on 100 mm cell-culture dishes. After allowing sufficient time for adhesion and spreading, cells were serum-starved overnight with serum-free cell-culture media DMEM/F-12 supplemented with insulin-transferrin-selenium (ITS). LOXL2 full-length and mutant LOXL2 proteins were transiently overexpressed using Adenoviral vectors (MOI = 25, 24 h), followed by addition of FXa (1 µg/ul) with or without rivaroxaban (100 nM) for 24 h. Full-length and processed forms of secreted LOXL2 in the media and ECM were then determined by Western blotting. Soluble LOXL2 in the media was used to detect total activity using the fluorimetric assay.

### Western blotting

Adherent cells were rinsed in PBS (Thermo Fisher; KH_2_PO_4_ 1.06 mM, NaCl 155.2 mM, Na_2_HPO_4_−7H_2_O 2.97 mM, pH7.4) twice, following which cells were lysed with Mammalian protein extraction reagent (M-PER; 300 µl; Thermo Fisher) containing protease inhibitors (Roche). Soluble proteins and insoluble matrix fraction were separated by centrifugation (10,000 × *g* for 15 minutes) at 4 °C. Protein concentration in the soluble fraction was determined using the Bradford assay (BioRad Protein Assay reagent). For soluble proteins, equal amounts of protein were withdrawn, boiled with Laemmli buffer, and loaded on gels. The insoluble matrix fraction was directly resuspended in 1.5× Laemmli buffer in a normalized volume based on cytosolic protein concentration (100 μl per mg soluble protein) and boiled. Equal volumes of the ECM fraction were then loaded on gels. Conditioned media was collected and centrifuged to eliminate cell debris. Protein from 1.5 ml of media was enriched by adding 10 µl StrataClean Resin (Agilent), rocking for 5 minutes, followed by centrifugation at 8,000 × *g* for 5 minutes. Supernatant was discarded and StrataClean resin pellet was resuspended in 50 μl 1.5× Laemmli buffer and boiled for 15 min. A normalized volume, based on cytosolic protein concentration, was loaded on gels.

Protein samples were fractionated by SDS-PAGE and electro-transferred onto nitrocellulose membrane. Membranes were blocked in 3% nonfat milk in TBST (Tris-buffered saline, 0.1% Tween 20) and then incubated with primary antibody (1:1000, 2 h), washed three times with TBST, and incubated with secondary antibody (1:10,000, 2 h). Membranes were washed in TBST and developed with the Clarity Western ECL system (Bio-Rad).

### Fluorimetric LOX activity assay

Lysyl oxidase (LOX) activity was measured using Amplite Fluorimetric Lysyl Oxidase Assay Kit (AAT Bioquest), following vendor’s protocol. Briefly, cells were lysed in M-PER buffer, and soluble cytosolic proteins were recovered by centrifugation, and protein concentration was determined using the Bradford assay (BioRad). Cell-culture media was concentrated 20-fold by centrifugal filtration (10 K molecular weight cutoff; Amicon Ultra centrifugal filter units; Millipore). 50 µL of sample (soluble cytosolic proteins or concentrated conditioned cell-culture medium) was added to 50 µL of LOX working solution containing LOX substrate, horseradish peroxidase, and Amplite horseradish peroxidase substrate. The samples were incubated at 37 °C and fluorescence was measured at Ex/Em = 540/590 nm every 5 minutes for a total of 1 h (SpectraMax Gemini). The slope of fluorescence intensity over time was calculated and normalized to the protein concentration determined in the soluble cytosolic fraction.

### Protein extraction from mouse organs

Male and female C57Bl/6 J mice aged 12–14 weeks were used in this study with appropriate Institutional Animal Care and Use Committee approvals. All vertebrate animal experiments were performed in accordance with relevant guidelines and regulations. All animal protocols were approved by the Institutional Animal Care and Use Committee of Johns Hopkins University. Mice were maintained in the Johns Hopkins University School of Medicine pathogen-free animal care facility on a 12-h dark/light cycle. Animals were fed and watered ad libitum. Mouse organs (liver, heart, kidney, and aorta) were pulverized under liquid nitrogen, then homogenized and sonicated in M-PER buffer containing protease inhibitors. Soluble proteins and insoluble matrix were separated by centrifugation at 10,000 × *g* for 15 minutes. Bio-Rad protein assay reagent was used to determine protein concentration in the soluble fraction. Western blotting was performed using 25 µg of the soluble fraction.

### Factor Xa mediated processing of LOXL2 mutants to identify processing site

HEK293 cells were seeded at 60% confluence in DMEM with 10% FBS. After 24 h, media was replaced with reduced serum medium OPTI-MEM (Gibco). Cells were transfected using Lipofectamine 3000 reagent kit (Thermo Fisher) with pEZY LOXL2 wild-type (WT), S300P-LOXL2, R316G/K317E-LOXL2, and R338G/V339P-LOXL2. Empty vector was added to control cell samples. After 24 h, media was replaced with serum-free DMEM/F-12. Media was collected 72 h post transfection, and incubated with and without FXa (1 μg/ml, 37 °C, gentle rocking) for 2 h. Proteins in media were enriched with StrataClean beads and LOXL2 processing was detected by Western blotting. For each mutant, LOXL2 processing was quantified as the percent loss of full length LOXL2 in the FXa containing sample vs untreated control.

### In situ LOXs activity in VSMCs

Assay procedure was performed as previously described with minor modifications^32^. Briefly, A7r5 cells seeded on coverslips at 80% confluence were allowed to adhere, then serum-starved overnight in DMEM/F-12 supplemented with ITS. LOXL2, ΔN-LOXL2, or LOXL2-DM was overexpressed by adenoviral transduction (MOI = 25; 24 h), following which the activity assay was initiated. To measure the effect of FXa processing on LOXL2 activity, in a subset of coverslips with LOXL2 overexpression, FXa (1 µg/ul) was added with or without rivaroxaban (100 nM). In another subset of coverslips with LOXL2 overexpression, inhibitors were added as follows: PAT-1251 only (10 µM; LOXL2-specific inhibitor), or BAPN (10 µM) + PAT-1251 (10 µM). BHZ (100 μM) was then delivered to confluent monolayers of cells for 24 h. Samples were then gently rinsed free of excess BHZ twice with sterile PBS (rocking followed by suctioning off the PBS). Cells were then fixed in 3.7% formaldehyde for 30 minutes before proceeding to fluorescence staining.

### Fluorescence staining

Cells were cultured on tissue culture coverslips, treated as indicated, and fixed, but not permeabilized. Fixed samples were blocked in 3% bovine serum albumin (BSA, Sigma-Aldrich) in PBS for 1 h. LOXL2 and ECM substrates were labeled by incubating the samples with a mixture of two primary antibodies raised in different species (1:150 in 1.5% BSA, 2 h) followed by incubation with a mixture of corresponding secondary antibodies (1:250 in 1.5% BSA, 2 h) in the dark. Following the in situ LOXs activity assay, biotinylated allysine hydrazones were detected by fluorescein (DTAF) streptavidin (1:250, 2 h). Nuclei were labeled with DAPI (1 μg/mL, 15 min). Between each step, coverslips were gently rinsed with PBS (3 times, 5 minutes each). All staining procedures were performed at room temperature in humidity chambers. Coverslips were mounted on slides with DAKO mounting medium (Agilent) and sealed. Confocal images for immunostained samples were taken using Leica SP8 confocal microscope and at least 5 non-overlapping images were obtained for each specimen. Fluorescence intensity was measured as mean gray value using Image J, and calculated as a percentage of the average intensity of control samples.

### Proximity ligation assay (PLA)

PLA was performed with Duolink® In Situ Red Starter Kit Mouse/Rabbit Reagents (DUO92101-1KT) following manufacturer’s protocol. Briefly, cells were cultured on coverslips, treated as indicated, and then fixed, but not permeabilized. Coverslips were incubated with the Blocking Solution for 1 h, followed by incubation with a mixture of the two primary antibodies raised in mouse and rabbit (1:150 in Antibody Diluent, 2 h). Coverslips were then incubated with two PLA secondary probes, anti-mouse MINUS and anti-rabbit PLUS diluted 1:5 in the Antibody Diluent for 1 h. Secondary probes were then ligated with provided ligase for 30 minutes, and amplified by incubation with the Amplification buffer and polymerase in the dark for 100 minutes. All staining incubations were performed at 37 °C in humidity chambers. Samples were rinsed twice for 5 min each in 1× Wash Buffer A between above steps, and rinsed twice for 10 min each in 1× Buffer B followed by a 1 min 0.01× Buffer B wash for the final washes. Finally, coverslips were mounted using a minimal volume of Duolink® In Situ Mounting Medium with DAPI.

### In vitro binding assay

C-terminus 6xHis-tagged full-length wild-type LOXL2 was overexpressed in A7r5 cells by adenoviral transduction. The conditioned media containing overexpressed LOXL2-6xHis was collected and used. This media contains both full-length and processed forms of LOXL2 due to endogenous FXa activity. The media was split into three parts to obtain the following three groups: (1) control, (2) FXa (1 μg/ml), and (3) FXa (1 μg/ml) + rivaroxaban (100 nM). After 2 h of incubation at room temperature, the full length and processed C-terminus 6xHis-tagged LOXL2 from each of these parts was immobilized onto agarose beads using anti-His antibody. Unbound conditioned media proteins were then removed by rinsing the beads three times with ice-cold PBS. Beads were then incubated with a mixture of 10 ng/mL of each COLI and COLIV proteins for 2 h at room temperature with gentle mixing. Beads were washed 3× in PBS to remove unbound collagens. Bound proteins were eluted by boiling in 1× Laemmli buffer (100 μl). COLI and COLIV binding to LOXL2 was then determined by Western blotting of equal volumes of the eluate.

### Gene expression analysis

RNA was extracted from A7r5 cells using column Aurum™ Total RNA Mini kit (Bio-Rad) and reverse transcribed using iScript™ cDNA Synthesis Kit (Bio-Rad). The expression of *LOX, LOXL1, LOXL2, LOXL3, COLI*, and *COLIV* genes were analyzed by real-time PCR. *GAPDH* was used as housekeeping control. Primers used are shown in Table 2. The relative expression of these genes was calculated using the ΔΔCt method, with untreated A7r5 cells as the control.

**Table 2:** List of qPCR primers.

*LOX*	Fw	ACTTCCAGTACGGTCTCCCGGAC
	Rv	TCATAGTCTCTGACATCCGCCCT
*LOXL1*	Fw	TGTATTCCTTGCGTTGTGCG
	Rv	TGGTGACAGCTATGCCACTC
*LOXL2*	Fw	GGCTATGTGGAGGCCAAGTCCT
	Rv	CCACACCAACATCTTCAGTGTGC
*LOXL3*	Fw	CCGGTTGGACCCACAGT
	Rv	GCACATTCAGTCACACTTCC
*GAPDH*	Fw	AGGCTGAGAATGGGAAGCTG
	Rv	TACTCAGCACCAGCATCACC
*COL I*	Fw	GTACATCAGCCCAAACCCCA
	Rv	TCGCTTCCATACTCGAACTGG
*COL IV*	Fw	TCCTCACTGTGGATTGGCTA
	Rv	CGATGAATGGGGCACTTCTA

### Statistics and reproducibility

Each experiment was conducted in at least five biological replicates. ImageLab (version 6.0.1) was used for western blot densitometry analysis. Statistical analyses were performed using GraphPad Prism (version 8.0.1). Results are shown as mean ± standard error of the mean (SEM), unless otherwise stated. To compare the two means, Student’s *t* test was used. To compare more than two means, ordinary one-way ANOVA was used with Bonferroni post hoc analysis. *P* values below 0.05 were considered significant and are indicated in the graphs.

### Reporting summary

Further information on research design is available in the Nature Portfolio Reporting Summary linked to this article.

## Supplementary information

Supplementary Figures

Description of Additional Supplementary Data

Supplementary Data 1

Reporting Summary

## Data Availability

The source data for graphs are available in Supplementary data 1. Uncropped Western blot images are available in Supplementary Fig. 5. Plasmids for S300P-LOXL2 (AddGene ID 200061), ΔN-LOXL2 (AddGene ID 200062), and R338G/V339P-LOXL2 (AddGene ID 200063) are available from Addgene.
