# Supplementary material for: Lysyl oxidase-like 2 processing by factor Xa modulates its activity and substrate preference
Source: Commun Biol. 2023 Apr 7;6:375. doi: 10.1038/s42003-023-04748-8 (PMC10082071; doi:10.1038/s42003-023-04748-8)

Supplementary Figures

Supplementary Figure 1: LOXL2 over-expression does not affect endogenous FXa. Representative Western blot of LOXL2 and FXa with and without adenoviral transduction of LOXL2. Blots are representative of 3 independent experiments.

Supplementary Figure 2: LOXL2 processing by FXa in the media of A7r5 cells overexpressing LOXL2. Blot is representative of 6-8 independent experiments.

Supplementary Figure 3. Representative Western blots.

a Representative Western blot of collagen I and collagen IV with and without FXa treatment. Data are representative of 3 independent experiments. FXa does not process type I collagen and type IV collagen.

b Representative Western blot showing LOXL2 processing in the cell culture supernatant (conditioned media) of HASMCs. Data are representative of 3 independent experiments.

Supplementary Figure 4. LOXL2 promotes LOX trafficking to the ECM.

Representative Western blot of LOXL2 and LOX in the media and cell-derived ECM of HASMCs. WT represents unmodified HASMCs, LOXL2KO represents HASMCs in which *LOXL2* gene was targeted by CRISPR-Cas9 gene editing. (n = 3 independent experiments).

Supplementary Figure 5. Uncropped Western blot images.

Supplementary Figure 1

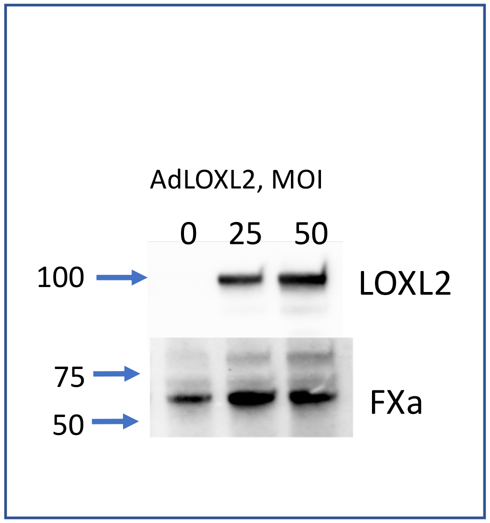

Supplementary Figure 2

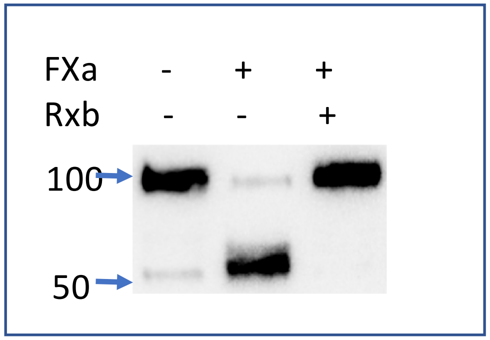

Supplementary Figure 3

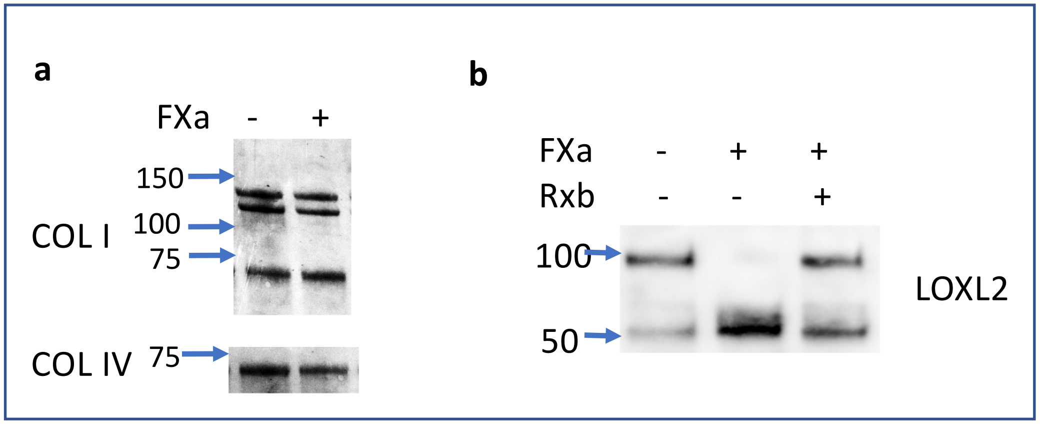

Supplementary Figure 4

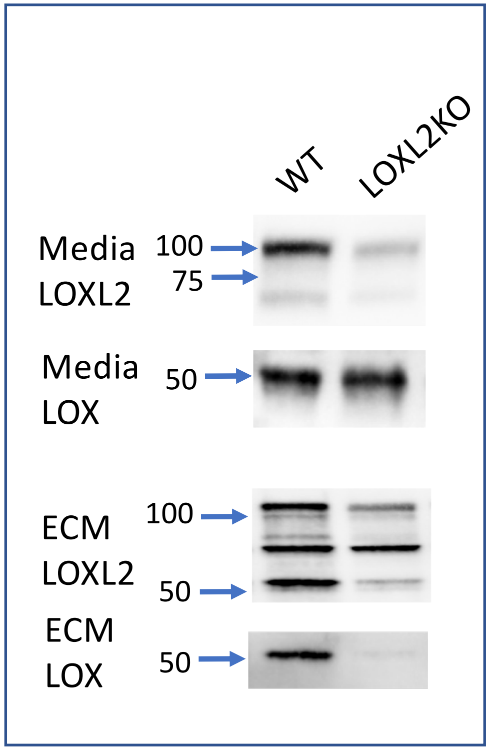

Supplementary Figure 5

Fig 1b

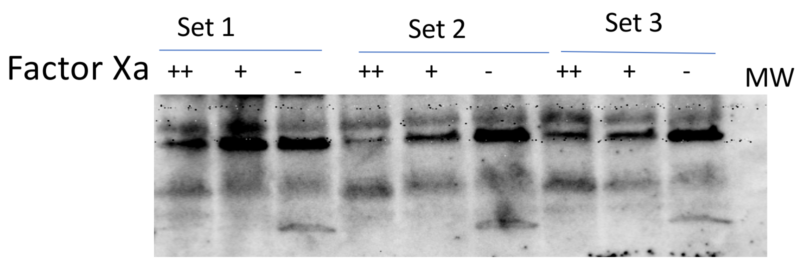

Fig 1c

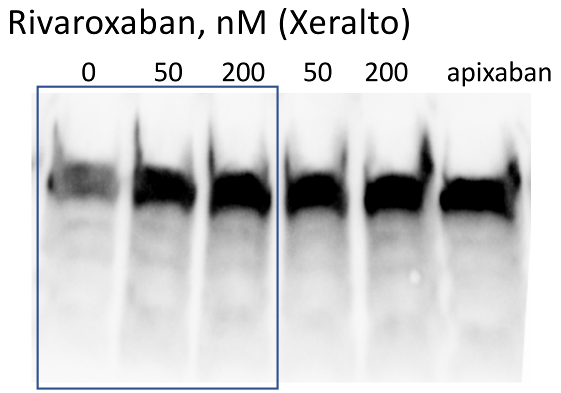

Figure 2a

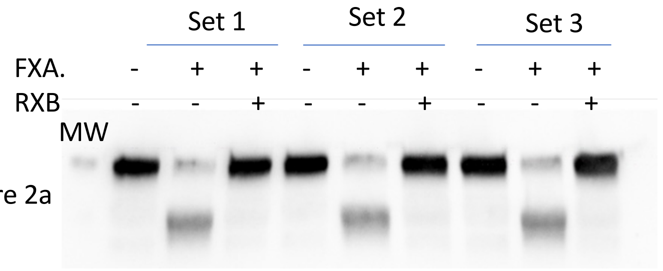

Figure 2b

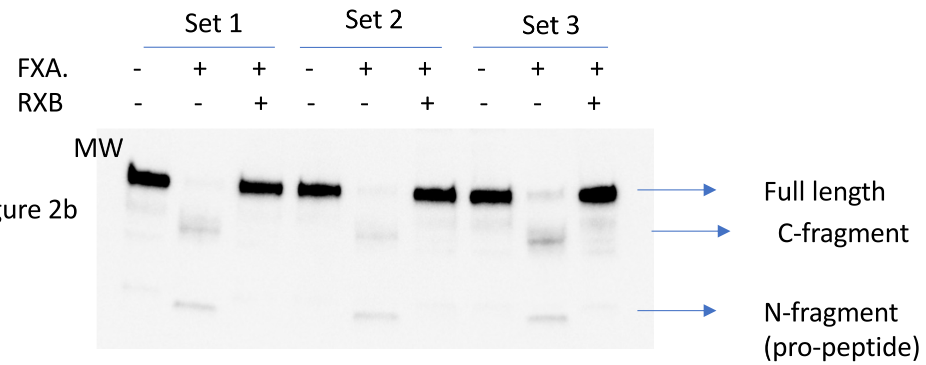

Supplementary Figure 5

Figure 4b

MW

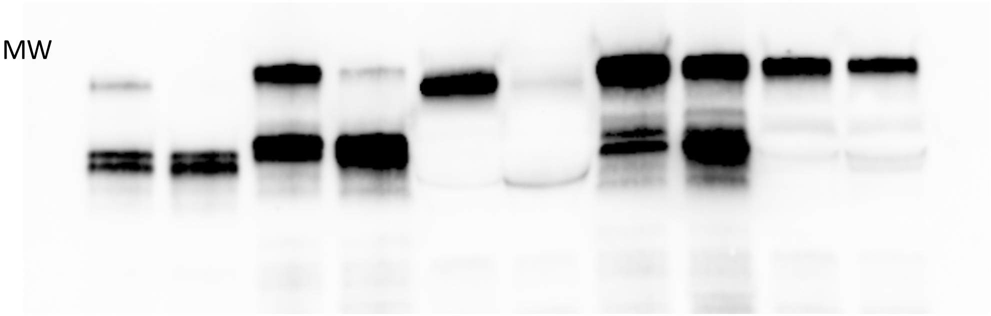

Figure 5bii

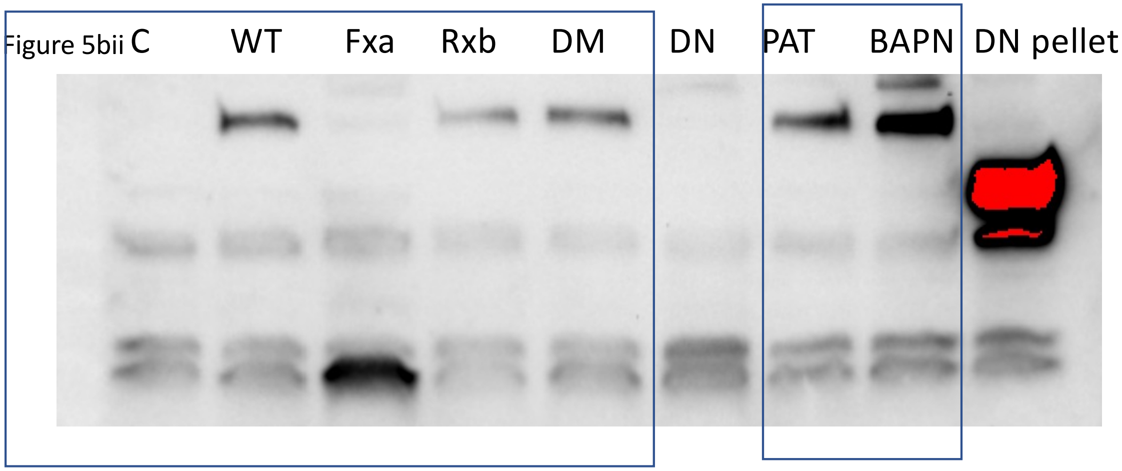

Lanes shown in Fig 5bii
Are in boxes.

Supplementary Figure 5

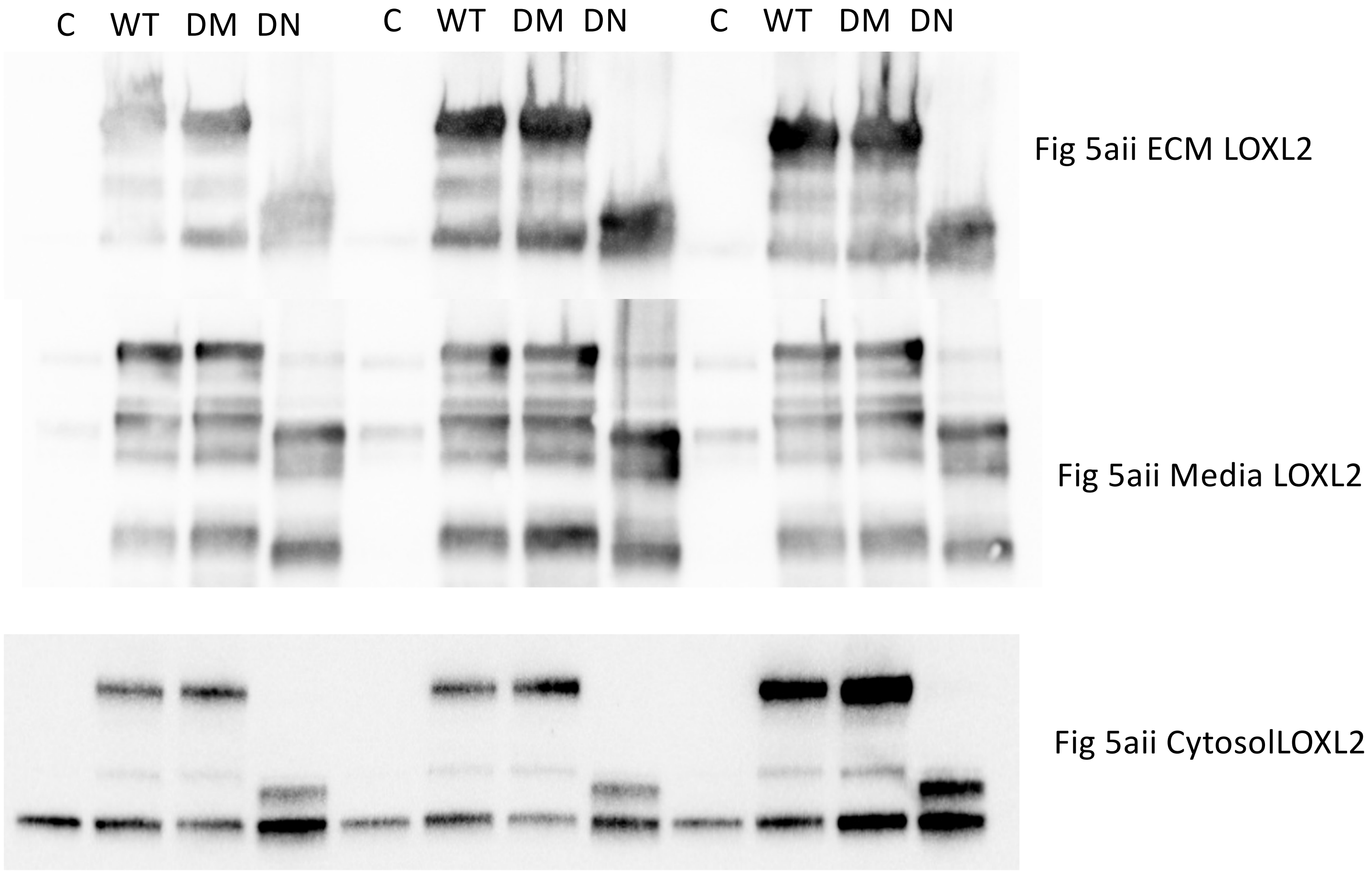

Supplementary Figure 5

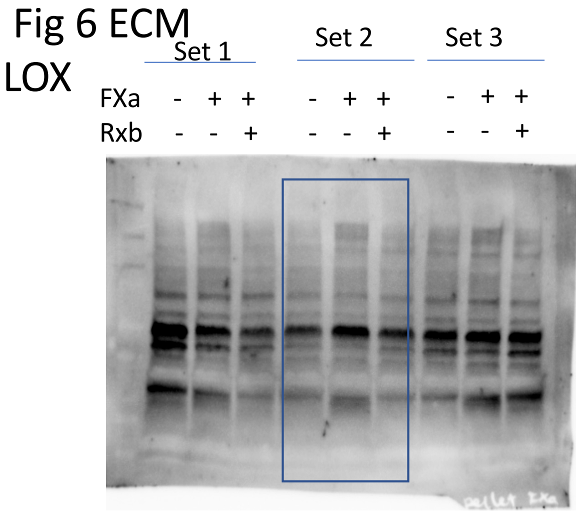

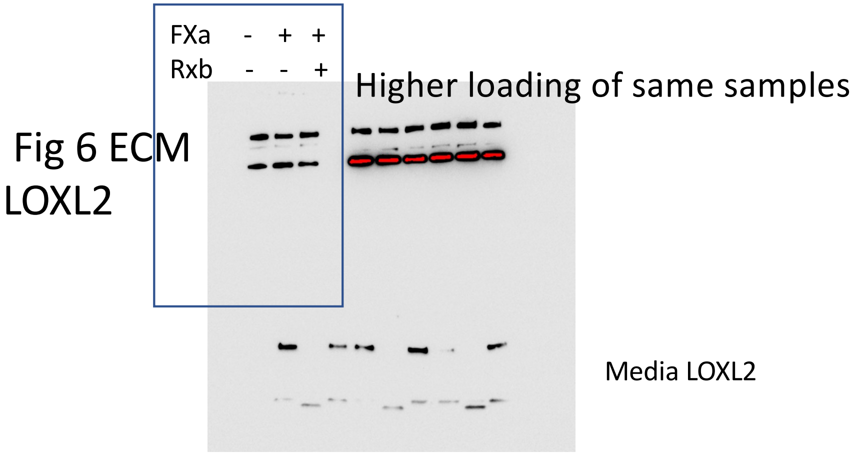

Supplementary Figure 5

Set 1 | Set 2

C. FL. FL+Fxa. Rxb. DM Coll C. FL. FL+Fxa. Rxb. DM. coll

Fig 7 cii

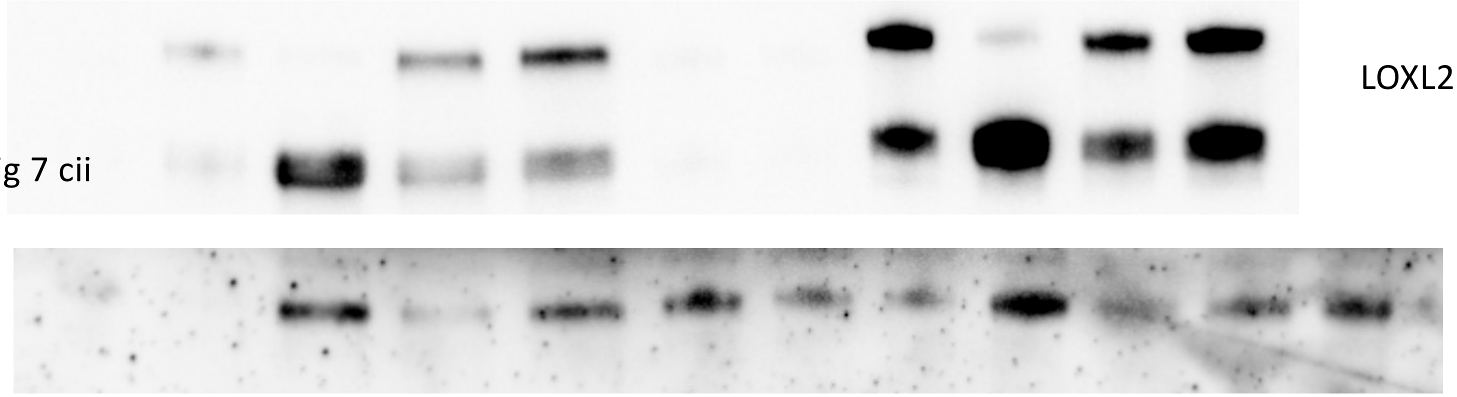

LOXL2

COLI

C = control beads LOXL2 Ab; no LOXL2

Set 2 | Set 1

FL. FL+Fxa. Rxb DM. COLIV. C

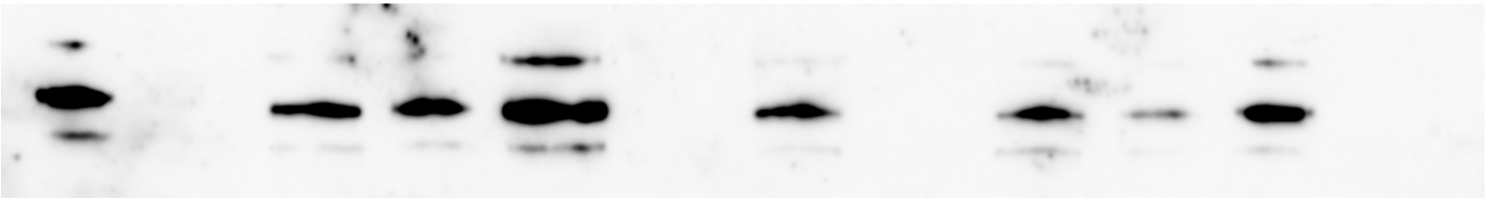

COLIV

Supplementary Figure 5

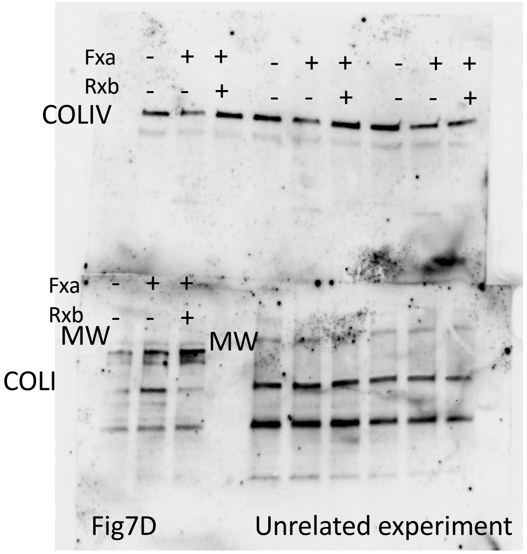

Fig 7di- ECM

Fig 7di- ECM

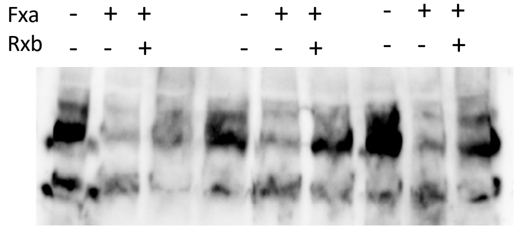

Fig 7di – ECMLOXL2

Figure 7d ii COLI

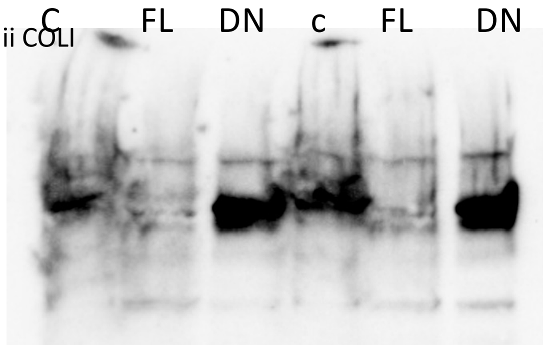

Figure 7d ii LOXL2

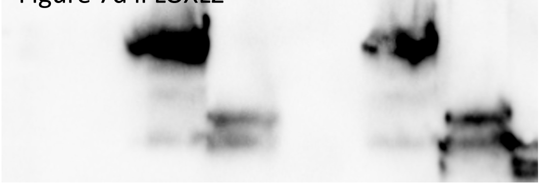

Figure 7d ii COLIV

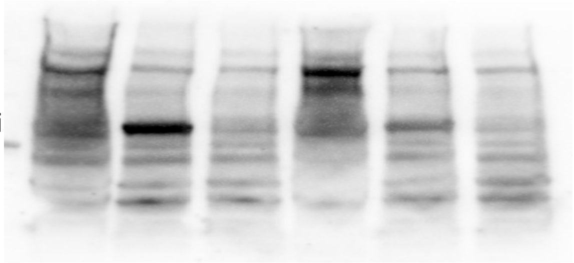

Supplementary Figure 5

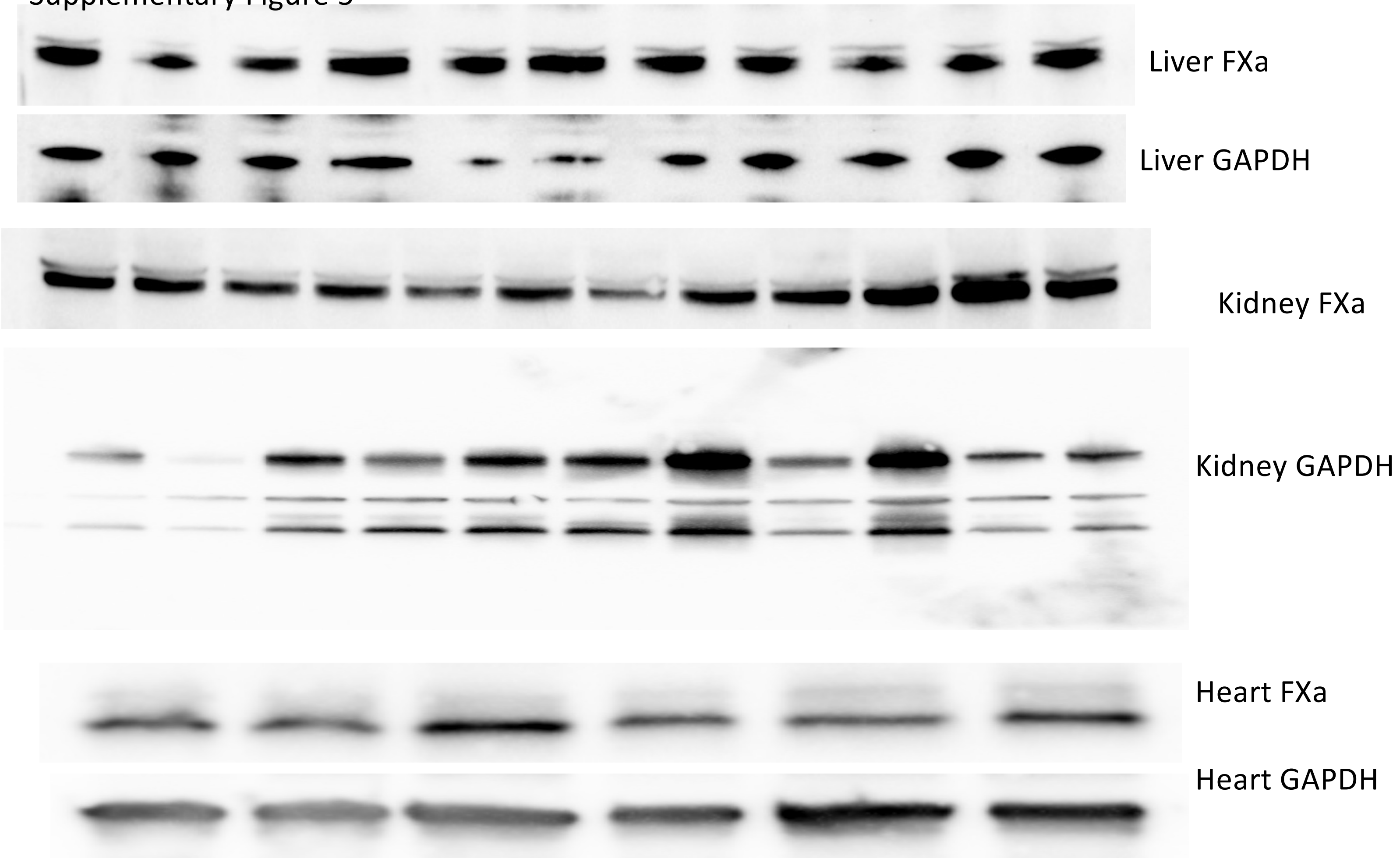

Supplementary Figure 5

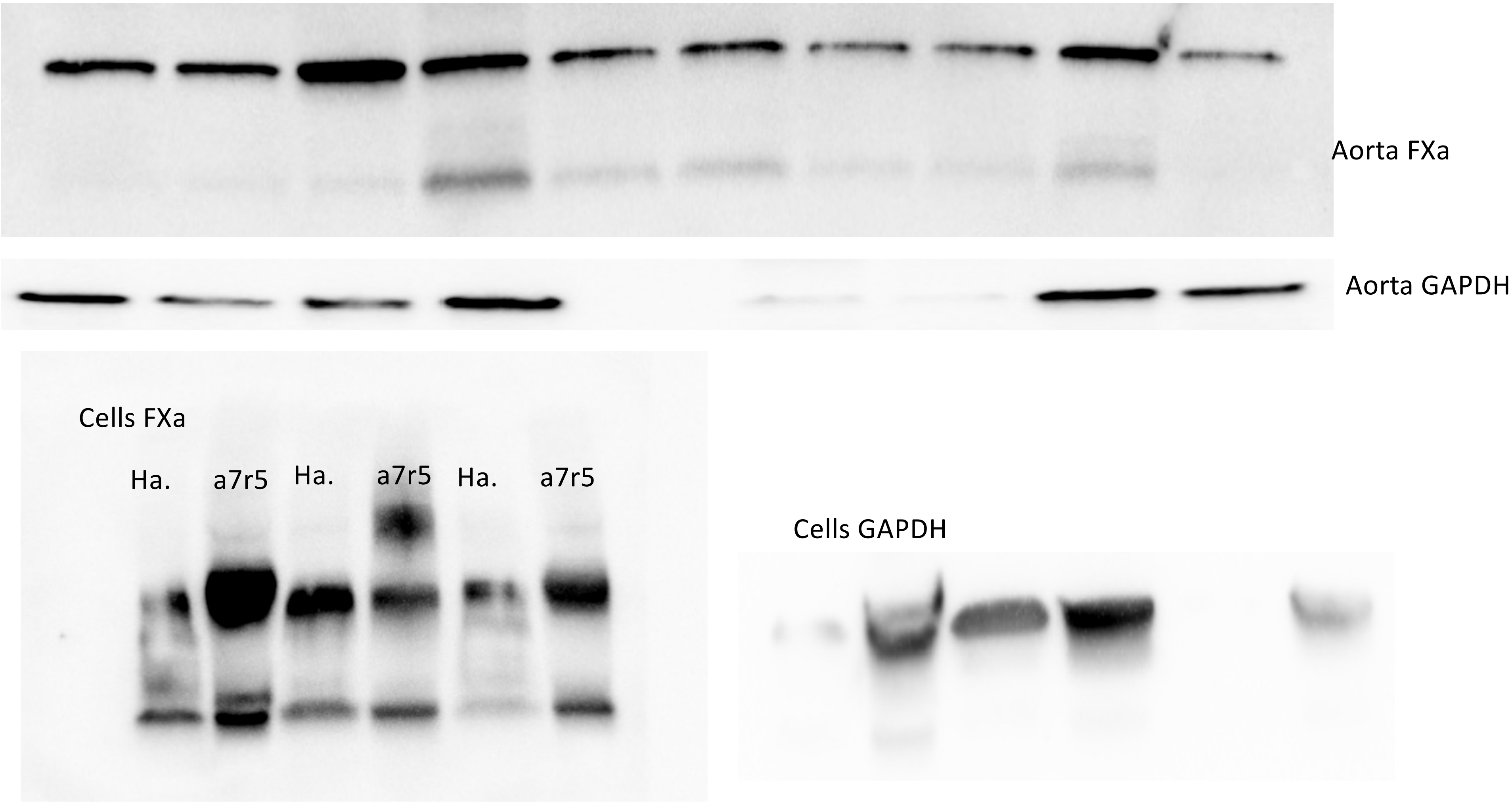

Supplement: Supplementary file 1 — Supplementary Figures [file 42003_2023_4748_MOESM1_ESM.pdf]
