# Supplementary material for: Lysyl oxidase-like 2 processing by factor Xa modulates its activity and substrate preference
Source: Commun Biol. 2023 Apr 7;6:375. doi: 10.1038/s42003-023-04748-8 (PMC10082071; doi:10.1038/s42003-023-04748-8)
Supplement: Supplementary file 2 — Description of Additional Supplementary Data [file 42003_2023_4748_MOESM2_ESM.docx]

**Description of Additional Supplementary Files**

**File name:** Supplementary Data 1

**Description:** : Excel spreadsheet of source data behind the graphs in the paper

**File name:** Supplementary Data 2

**Description:** Full western blots
