# Supplementary material for: Lysyl oxidase-like 2 processing by factor Xa modulates its activity and substrate preference
Source: Commun Biol. 2023 Apr 7;6:375. doi: 10.1038/s42003-023-04748-8 (PMC10082071; doi:10.1038/s42003-023-04748-8)
Supplement: Supplementary file 4 — Reporting Summary [file 42003_2023_4748_MOESM4_ESM.pdf]

nature portfolio

Corresponding author(s): Lakshmi Santhanam

Last updated by author(s): 3/08/2023

Reporting Summary

Nature Portfolio wishes to improve the reproducibility of the work that we publish. This form provides structure for consistency and transparency in reporting. For further information on Nature Portfolio policies, see our Editorial Policies and the Editorial Policy Checklist.

Statistics

For all statistical analyses, confirm that the following items are present in the figure legend, table legend, main text, or Methods section.

n/a	Confirmed	
☐	☒	The exact sample size (*n*) for each experimental group/condition, given as a discrete number and unit of measurement
☐	☒	A statement on whether measurements were taken from distinct samples or whether the same sample was measured repeatedly
☐	☒	The statistical test(s) used AND whether they are one- or two-sided<br>*Only common tests should be described solely by name; describe more complex techniques in the Methods section.*
☐	☒	A description of all covariates tested
☐	☒	A description of any assumptions or corrections, such as tests of normality and adjustment for multiple comparisons
☐	☒	A full description of the statistical parameters including central tendency (e.g. means) or other basic estimates (e.g. regression coefficient) AND variation (e.g. standard deviation) or associated estimates of uncertainty (e.g. confidence intervals)
☐	☒	For null hypothesis testing, the test statistic (e.g. *F*, *t*, *r*) with confidence intervals, effect sizes, degrees of freedom and *P* value noted<br>*Give P values as exact values whenever suitable.*
☒	☐	For Bayesian analysis, information on the choice of priors and Markov chain Monte Carlo settings
☒	☐	For hierarchical and complex designs, identification of the appropriate level for tests and full reporting of outcomes
☒	☐	Estimates of effect sizes (e.g. Cohen's *d*, Pearson's *r*), indicating how they were calculated

Our web collection on statistics for biologists contains articles on many of the points above.

Software and code

Policy information about availability of computer code

Data collection	LabChart, BioRad Image Lab, LasX confocal microscopy software,
Data analysis	Graph pad prism v9.0, BioRad Image Lab, Microsoft excel, FIJI (Image J).

For manuscripts utilizing custom algorithms or software that are central to the research but not yet described in published literature, software must be made available to editors and reviewers. We strongly encourage code deposition in a community repository (e.g. GitHub). See the Nature Portfolio guidelines for submitting code & software for further information.

Data

Policy information about availability of data

All manuscripts must include a data availability statement. This statement should provide the following information, where applicable:

- Accession codes, unique identifiers, or web links for publicly available datasets
- A description of any restrictions on data availability
- For clinical datasets or third party data, please ensure that the statement adheres to our policy

Data will be made available upon reasonable request

Human research participants

Policy information about studies involving human research participants and Sex and Gender in Research.

Reporting on sex and gender	NA
Population characteristics	NA
Recruitment	NA
Ethics oversight	NA

Note that full information on the approval of the study protocol must also be provided in the manuscript.

Field-specific reporting

Please select the one below that is the best fit for your research. If you are not sure, read the appropriate sections before making your selection.

☒ Life sciences ☐ Behavioural & social sciences ☐ Ecological, evolutionary & environmental sciences

For a reference copy of the document with all sections, see nature.com/documents/nr-reporting-summary-flat.pdf

Life sciences study design

All studies must disclose on these points even when the disclosure is negative.

Sample size	sample size was estimated to detect a 20% effect, alpha = 0.5, and power of 0.9
Data exclusions	data were excluded if 1) sampling error, 2) human error, or 3) measurement error.
Replication	Cell experiments were performed using at least 3 different lots of cells for HASMCs and from 3 different times of purchase for A7r5. For tissue experiments, samples were obtained from 2-3 males and 2-3 females.
Randomization	NA
Blinding	Data acquisition and data analysis were performed by different persons. Individual performing analysis was blinded to the treatment condition.

Reporting for specific materials, systems and methods

We require information from authors about some types of materials, experimental systems and methods used in many studies. Here, indicate whether each material, system or method listed is relevant to your study. If you are not sure if a list item applies to your research, read the appropriate section before selecting a response.

Materials & experimental systems

n/a	Involved in the study	
☐	☒	Antibodies
☐	☒	Eukaryotic cell lines
☒	☐	Palaeontology and archaeology
☐	☒	Animals and other organisms
☒	☐	Clinical data
☒	☐	Dual use research of concern

Methods

n/a	Involved in the study	
☒	☐	ChIP-seq
☒	☐	Flow cytometry
☒	☐	MRI-based neuroimaging

Antibodies

Antibodies used	LOX rabbit polyclonal (ThermoFisher PA1-46020), LOXL2 rabbit monoclonal C-terminal (Abcam ab179810), LOXL2 polyclonal (Abcam ab197779), COLI monoclonal antibody (Invitrogen MA1-26771), COLIV rabbit polyclonal (Assay Biotech C0157), Factor X/Xa polyclonal antibody (Invitrogen PA5-102412), GAPDH mouse monoclonal (Novus Bio NB300221), goat anti-mouse IgG (H+L)-HRP conjugate (Biorad 1706516), AffiniPure goat anti-rabbit IgG (H+L)-HRP conjugate (Jackson ImmunoResearch 111035144), Cy5 AffiniPure Goat Anti-Rabbit IgG (H+L) (Jackson ImmunoResearch, 111175144), Alexa Fluor 568 Goat anti-Rabbit IgG (H+L) Secondary

	Antibody (Invitrogen, A-11011), Alexa Fluor 488 Donkey anti-Mouse IgG (H+L) Secondary Antibody (Invitrogen, A-21202) and fluorescein (DTAF) streptavidin (Jackson ImmunoResearch, 016010084).
Validation	LOX and LOXL2 antibodies were validated by knockdown. FXa, CoII and COLIV abs were validated using purified proteins. Other abs were used based on prior publications and prior use in the lab.

Eukaryotic cell lines

Policy information about cell lines and Sex and Gender in Research

Cell line source(s)	A7r5 - ATCC; HEK293- ATCC<br>HASMC - Thermo
Authentication	Staining for SMC markers SMA, SM-22a, SM-MHC
Mycoplasma contamination	Cells were periodically tested for mycoplasma using a commercial kit.
Commonly misidentified lines<br>(See ICLAC register)	NA

Animals and other research organisms

Policy information about studies involving animals; ARRIVE guidelines recommended for reporting animal research, and Sex and Gender in Research

Laboratory animals	Mouse; C57Bl6
Wild animals	NA
Reporting on sex	NA
Field-collected samples	NA
Ethics oversight	Johns Hopkins University IACUC

Note that full information on the approval of the study protocol must also be provided in the manuscript.
